# The E3 ubiquitin ligase Cbl-b improves the prognosis of RANK positive breast cancer patients by inhibiting RANKL-induced cell migration and metastasis

**DOI:** 10.18632/oncotarget.4382

**Published:** 2015-06-08

**Authors:** Lingyun Zhang, Yuee Teng, Yibo Fan, Yan Wang, Wei Li, Jing Shi, Yanju Ma, Ce Li, Xiaonan Shi, Xiujuan Qu, Yunpeng Liu

**Affiliations:** ^1^ Department of Medical Oncology, The First Hospital of China Medical University, Shenyang, China

**Keywords:** breast cancer, RANK, Cbl-b, metastasis, migration

## Abstract

The receptor activator of nuclear factor κ-B ligand (RANKL)/RANK pathway plays an important role in breast cancer progression. Despite the known role of Casitas B-lineage lymphoma (Cbl)-b as an essential regulator of the RANKL/RANK pathway, its effect on RANK pathway in breast cancer remains unclear. Thus, the present study investigated the effect of Cbl-b on the prognosis of RANK-expressing breast cancer patients, as well as on RANKL/RANK pathway. The results showed that RANK and Cbl-b expression was separately detected in 154 (154/300, 51.3%) and 165 (165/300, 55.0%) breast cancer tissue samples. In RANK-expressing breast cancer patients, Cbl-b expression was correlated with low metastasis rate (*p* = 0.004), better disease-free survival (DFS) and breast cancer-specific survival (BCSS) (*p* = 0.004 and *p* = 0.036, respectively). In addition, multivariate analysis showed that Cbl-b expression was an independent predictor of DFS (*p* = 0.038). Animal experiment results demonstrated that silencing Cbl-b expression in breast cancer cells increased the incidence of lung metastasis in nude mice. Further mechanism investigation revealed that Cbl-b down-regulated RANK protein expression and inhibited RANKL-induced breast cancer cell migration by negatively regulating the Src-Akt/ERK pathway. Our results suggest that Cbl-b improves the prognosis of RANK-expressing breast cancer patients by inhibiting RANKL-induced breast cancer cell migration and metastasis.

## INTRODUCTION

Breast cancer is the most common cancer in women worldwide, and the occurrence of metastatic disease increases mortality and affects quality of life. Recent studies have shown that chemokine pathways play an important role in the development of distant metastasis from primary site. The receptor activator of nuclear factor κ-B ligand (RANKL)/receptor activator of nuclear factor κ-B (RANK) pathway, which is essential for osteoclast maturation and activation, also functions as a chemokine-like pathway. The RANK protein is expressed in osteoclasts [1], dendritic cells [2], T cells [3] and mammary epithelial cells [4], and recently, its expression in some cancer cells, especially in breast cancer cells, has been demonstrated [5-8]. RANKL promotes breast cancer cell migration and induces metastasis of breast cancer cells to the bone and lungs *in vitro* and in xenograft experiments [6, 9, 10], suggesting that the RANK pathway plays a role in breast cancer progression. However, whether RANK could be used as a biomarker of breast cancer progression is controversial [5, 11-14]. RANK expression was reported to be associated with a higher risk of relapse and death in breast cancer patients [11, 12]. However, in a different study, RANK mRNA expression was not associated with poor prognosis in breast cancer patients [13]. Another study even reported that high levels of RANK or RANKL mRNA expression were correlated with better overall survival [14]. These contradictory reports indicate that the role of the RANK pathway in breast cancer metastasis is complex and further investigation is necessary to clarify its effect.

The RANKL/RANK pathway involves several effectors including tumor necrosis factor (TNF) receptor-associated factor 6 (TRAF6), nuclear factor kappa-light-chain-enhancer of activated B cells (NF-κB), mitogen activated protein kinase (MAPK), phospho-inositide 3 kinase (PI3K)/Akt, and nuclear factor of activated T-cells (NFAT), and others. The expression of molecules downstream of the RANKL/RANK pathway, such as TRAF6, NF-κB and NFAT, is modulated by the ubiquitin-proteasome system (UPS) [15-19]. The UPS is a common and essential protein degradation pathway that regulates the stability and function of many proteins [20, 21]. Our previous study demonstrated that the UPS inhibitor bortezomib upregulates RANK expression and enhances RANKL-induced breast cancer cell migration [22], suggesting that the UPS is a negative regulator of the RANK pathway. Casitas B-lineage lymphoma (Cbl)-b is an essential enzyme in the UPS and functions as multifunctional adaptor protein or E3 ubiquitin ligase. In a previous study from our group, we showed that Cbl-b is expressed in gastric cancer, colon cancer, and breast cancer cells [23]. Cbl-b functions as a negative regulator of several signaling molecules including PI3k/Akt, ERK, and NF-κB in various cell types [17, 23-26], which is downstream of the RANKL/RANK pathway. Cbl-b suppresses epidermal growth factor (EGF) receptor-mediated epithelial cell migration [27], and promotes SDF-1/CXCL12-induced T cell migration [28]. However, the effect of Cbl-b on RANKL induced breast cancer cell migration is unclear, and whether it plays a role in the prognosis of RANK-expressing breast cancer patients remains to be elucidated.

In the present study, we demonstrated that Cbl-b expression was a predictor of favorable prognosis in RANK-expressing breast cancer patients. Cbl-b suppressed RANK expression and inhibited RANKL-induced breast cancer cell migration and metastasis through the negative regulation of the Src/Akt and Src/ERK pathways. Our results provide new insight into the regulatory mechanism of RANKL/RANK pathway-mediated breast cancer cell migration, and suggest that combined analysis of Cbl-b and RANK as biomarkers could be useful for the characterization of breast cancer patients.

## RESULTS

### Cbl-b predicts better prognosis in RANK-expressing breast cancer patients

#### The prognostic value of RANK in breast cancer patients

**Figure 1:**
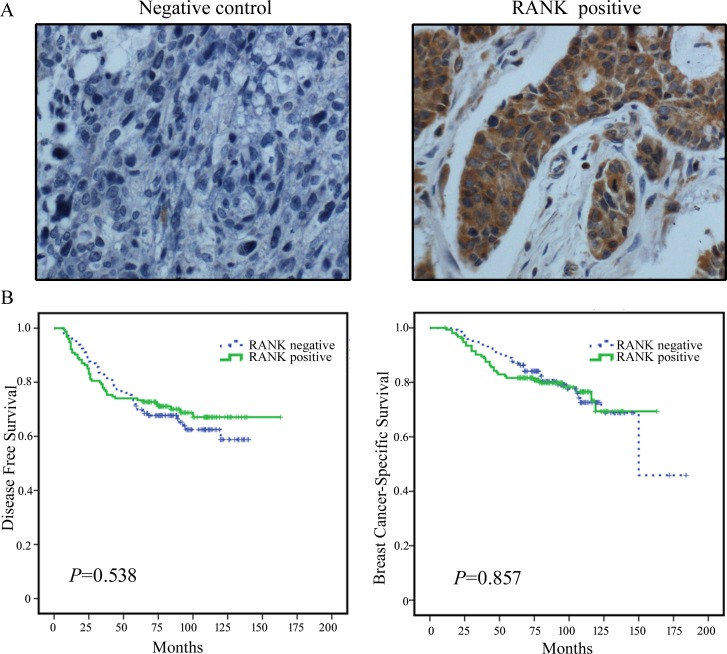
Representative images of RANK immunohistochemical staining in breast cancer tissues and the prognostic value of RANK in breast cancer patients **A.** Representative images of RANK immunohistochemical staining of breast cancer tissues. Negative control and positive staining is shown in breast cancer tissues. RANK positive staining is observed in the cell membrane and cytoplasm (brown). Magniﬁcation ×400. **B.** Kaplan-Meier survival curves for disease-free survival (DFS) and breast cancer-specific survival (BCSS) in RANK-negative patients (*n* = 146) and RANK-positive patients (*n* = 154).

A total of 300 histologically conﬁrmed breast cancer samples were obtained, including invasive ductal carcinomas, invasive lobular carcinomas and carcinomas of another type. The median age of patients was 51 (range 26–76) years, and the median follow-up time was 84 (range11–184) months. A total of 99 (33.0%) patients developed disease progression, and 68 (22.7%) patients died of disease progression. RANK expression was evident upon immunostaining (Figure 1A). Positive RANK expression was observed in 154 (51.3%) breast cancer tissue samples. The RANK-positive expression rate was significantly higher in the HER2-positive than in the HER2-negative population (*p* = 0.016, Supplementary Table S1, available online).

The prognostic value of RANK in breast cancer patients was examined by performing survival analyses. The results showed no differences in DFS (*p* = 0.538) and BCSS (*p* = 0.857) between RANK-positive and RANK-negative patients (Figure 1B). The fact that RANK expression was not correlated with patient prognosis was not in accordance with our expectations and indicated that other factors may affect the prognosis of RANK-expressing breast cancer patients.

#### The prognostic value of Cbl-b in RANK positive breast cancer patients

**Figure 2:**
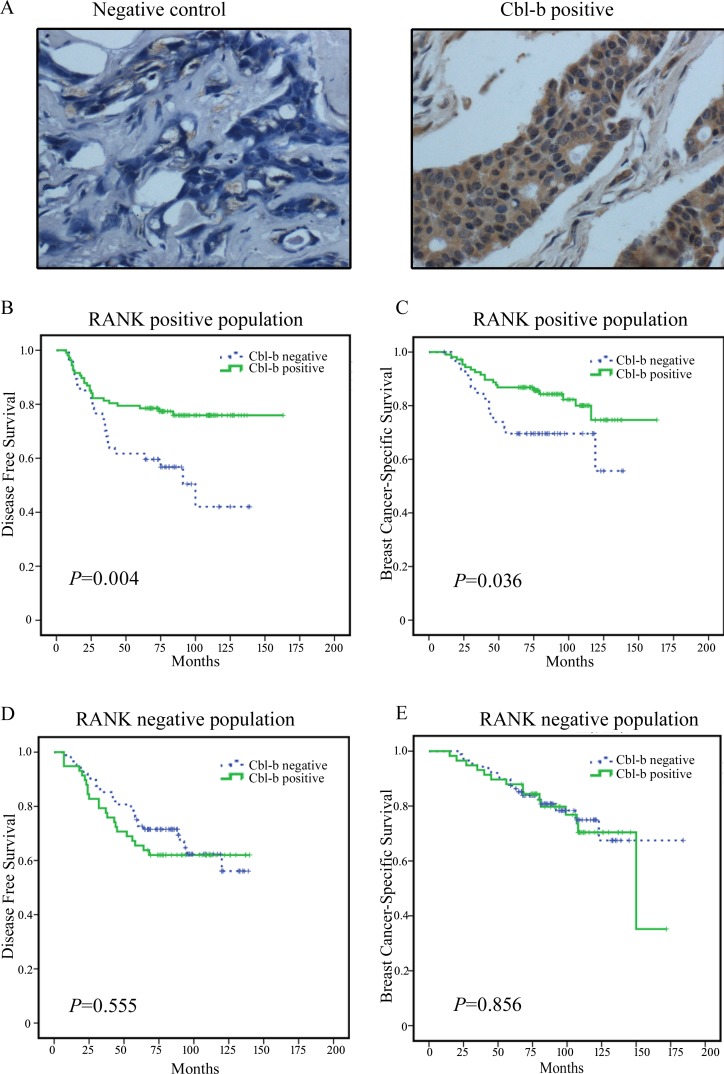
Representative images of Cbl-b immunohistochemical staining in breast cancer and the correlation between Cbl-b expression and patient survival in RANK positive breast cancer patients **A.** Representative images of Cbl-b immunohistochemical staining in breast cancer tissues. Negative control and positive staining is shown in breast cancer tissues. Cbl-b positive staining is observed in the cytoplasm (brown). Magniﬁcation × 400. **B.**-**E.** Kaplan-Meier survival curves for DFS **B.** and BCSS **C.** in RANK positive patients (*n* = 154) including Cbl-b negative patients (*n* = 47) and Cbl-b positive patients (*n* = 107). In RANK negative patients (*n* = 146), no significant differences in DFS **D.** and BCSS **E.** were observed between Cbl-b negative patients (*n* = 88) and Cbl-b positive patients (*n* = 58).

Since Cbl-b plays an essential role in the RANKL/RANK pathway in osteoclasts, next, we examined the prognostic value of Cbl-b in RANK-positive breast cancer patients. Positive Cbl-b expression was detected in 165 (55.0%) of 300 histologically confirmed breast cancer samples (Figure 2A) and in 107 (69.5%) of 154 RANK-positive breast cancer samples. In RANK positive breast cancer tissue samples, Cbl-b expression was signiﬁcantly lower in patients who developed metastasis than in those that did not (*p* = 0.004, Table 1). The 47 metastatic patients included 35 patients with visceral metastasis, 12 patients with non-visceral metastasis, 22 patients with bone metastasis, and 25 patients with non-bone metastasis. Survival analysis of RANK-positive patients showed that positive Cbl-b expression was significantly associated with favorable prognosis in terms of DFS (Figure 2B) (*p* = 0.004) and BCSS (*p* = 0.036) (Figure 2C) compared to negative Cbl-b expression. The median DFS of Cbl-b negative and Cbl-b positive patients was 73 and 82 months, respectively (hazard ratio (HR) = 0.445, 95% confidence interval (CI) 0.250–0.791). However, in RANK negative patients, no difference in DFS (*p* = 0.555) (Figure 2D) or BCSS (*p* = 0.856) (Figure 2E) was observed between Cbl-b positive and negative patients.

**Table 1:** Correlation of Cbl-b expression with clinic-pathological parameters in 154 RANK positive breast cancer patients

Clinic-pathological parameters	n	Cbl-b expression† (%)	p
**Histological type**			0.650
**invasive ductal carcinomas**	137	96(70.1)	
**invasive lobular carcinoma and others**	17	11(64.7)	
**Age (years)**			0.495(exact)
**≤35**	10	6(60.0)	
**>35**	144	101(70.1)	
**Tumor size(cm)**			0.137
**≤2**	49	38(77.6)	
**>2**	105	60(65.7)	
_P_**N stage**			0.063
**0**	63	49(77.8)	
**1-3**	91	58(63.7)	
**Histology grade**			0.064(exact)
**I**	14	13(92.9)	
**II+III**	123	82(66.7)	
**ER/PR status**^††^			0.457
**Negative**	74	49(66.2)	
**Positive**	78	56(71.8)	
**Unknown**	2		
**HER2 status**^§^			0.985
**Negative**	88	62(70.5)	
**Positive**	64	45(70.3)	
**Unknown**	2		
**Triple negative**			0.281
**no**	116	83(71.6)	
**yes**	31	23(62.2)	
**Unknown**	1		
**Metastasis**			**0.004**
**no**	107	82(76.6)	
**yes**	47	25(53.2)	

*P* values shown in bold are statistically significant (two-sided, *p* < 0.05)

† Cbl-b expression was classified as “negative”: arbitrary scale=0-1, and “positive”: arbitrary scale= 2-6, as described in materials and methods.

†† ER/PR status: Estrogen receptor or progesterone receptor status by immunohistochemistry (IHC); negative: ER and PR double negative; positive: ER or PR positive.

§ HER2 status: HER2 positive status is IHC 3+ or fluorescence in situ hybridization (FISH) positive; HER2 negative status is IHC 0, 1+ or FISH negative, if. IHC 2+, FISH is applied to confirm the HER2 status.

Furthermore, Univariate analysis of the 154 RANK-positive patients showed that negative Cbl-b expression, age <35 years, tumor size >2 cm and lymph node metastasis predicted poor DFS and BCSS. Multivariate analysis showed that Cbl-b expression or age <35 years was an independent predictor for DFS, and age <35 years or tumor size >2 cm was an independent predictor for BCSS (Table 2).

Taken together, these data indicated that Cbl-b inhibited the incidence of distant metastasis and Cbl-b expression was a favorable predictor of prognosis in RANK-positive breast cancer patients.

**Table 2:** Cox univariate and multivariate analysis of disease-free survival (A) and disease-specific survival (B) in RANK positive breast cancer patients (*n* = 154)

Risk factors	Univariate analysis		Multivariate analysis	
	HR (95% CI)	*p*	HR(95% CI)	*p*
**A Disease-free survival**				
Cbl-b positive *versus* negative	0.445 (0.250-0.791)	**0.006**	0.539(0.301-0.966)	**0.038**
Age ≤35 years *versus* >35 years	2.911 (1.232-6.882)	**0.015**	2.381 (1.004-5.649)	**0.049**
Tumor size >2 cm *versus* <2cm	2.919 (1.307-6.518)	**0.009**	2.198 (0.967-4.999)	0.060
Lymph node metastasis positive *versus* negative	2.682 (1.364-5.272)	**0.004**	1.988 (0.992-3.984)	0.053
Histology grade2-3 *versus*1	2.894 (0.699-11.975)	0.143		
ER/PR positive *versus* negative	0.920 (0.519-1.631)	0.776		
HER2 positive *versus* negative	1.357 (0.765-2.408)	0.297		
**B Breast cancer-speciﬁc survival**				
Cbl-b positive *versus* negative	0.492 (0.249-0.969)	**0.040**	0.631 (0.317-1.256)	0.190
Age ≤35 years *versus* >35 years	4.647 (1.899-11.376)	**0.001**	4.035 (1.643-9.908)	**0.002**
Tumor size >2 cm *versus* <2cm	3.839 (1.352-10.905)	**0.012**	2.997 (1.039-8.642)	**0.042**
Lymph node metastasis positive *versus* negative	2.998 (1.305-6.888)	**0.010**	2.125 (0.909-4.967)	0.082
Histology grade 2-3 *versus*1	2.123 (0.506-8.904)	0.304		
ER/PR positive *versus* negative	0.994 (0.534-2.044)	0.905		
HER2 positive *versus* negative	1.550 (0.791-3.036)	0.202		

HR, hazard ratio; CI, confidence interval.

*p* values shown in bold are statistically significant (Two-sided, *p* < 0.05).

### Cbl-b inhibits RANKL-induced breast cancer cell migration and metastasis

**Figure 3:**
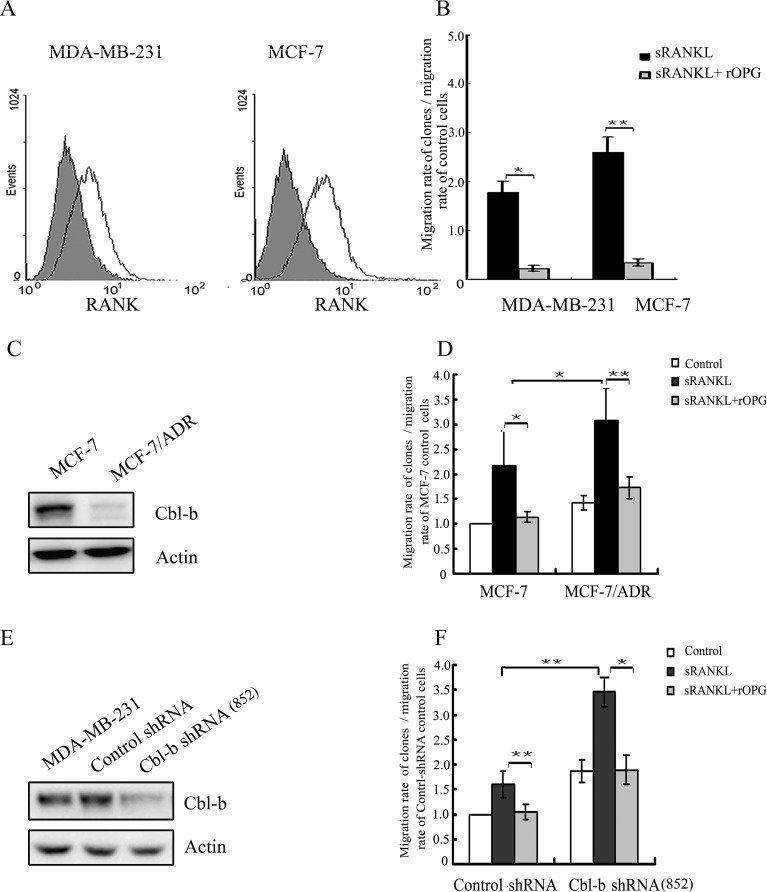
Cbl-b negatively regulated RANKL induced breast cancer cell migration **A.** RANK expression on the surface of MDA-MB-231 and MCF-7 cells was examined by ﬂow cytometry. Isotype control staining is shown in grey. **B.** MDA-MB-231 and MCF-7 cells were incubated with sRANKL (2 μg/ml) with or without rOPG (10 μg/ml), and in vitro migration activity was measured with the Transwell assay. Results are expressed as the mean ± SD. The data represent the results of at least three independent experiments. *p < 0.05, **p < 0.01 indicate significant differences compared with the control. **C.** Western blot analysis of Cbl-b protein levels in MCF-7 and MCF-7/ADR cells. **D.** MCF-7 and MCF-7/ADR cells were incubated with sRANKL (2 μg/ml) with or without rOPG (10 μg/ml), and in vitro migration activity was measured with the Transwell assay. *p < 0.05, **p < 0.01 indicate significant differences compared with the control. **E.** Western blot analysis showing the effect of shRNA against Cbl-b in MDA-MB-231 clones. **F.** MDA-MB-231 Cbl-b shRNA clones and control shRNA clones were incubated with sRANKL (2 μg/ml) with or without rOPG (10 μg/ml), and in vitro migration activity was measured with the Transwell assay. *p < 0.05, **p < 0.01 indicate significant differences compared with the control.

To explore the mechanism underlying the protective effect of Cbl-b against metastasis in RANK-expressing breast cancer, we performed functional studies. Previous studies from others group and ours [6, 29] showed that RANKL promotes the migration of breast cancer cells; therefore, we explored the role of Cbl-b in RANKL-induced breast cancer cell migration. Flow cytometry analysis showed that RANK was expressed on the cell surface of MDA-MB-231 and MCF-7 cells (Figure 3A). RANKL promoted the migration of breast cancer cells, and RANKL-induced cell migration was blocked with OPG (decoy receptor of RANKL) (Figure 3B). In a preliminary study, we showed that the breast cancer multi-drug resistance (MDR) cell line MCF-7/ADR, which is derived from the MCF-7 cell line by selection for growth in increasing concentrations of adriamycin, expresses lower levels of Cbl-b (Figure 3C) than MCF-7 cells. Additionally, Transwell migration assays showed that the migration rate of RANKL-induced MCF-7/ADR cells was significantly higher than that of MCF-7 cells (Figure 3D). To further evaluate the effect of Cbl-b on the RANKL/RANK pathway, we established MDA-MB-231 Cbl-b shRNA cell lines. Meanwhile, we also established Cbl-b overexpression cell lines by transfecting MCF-7 cells with pcDNA3.1 plasmid (Figure 3E, Supplementary Figure S1, available online). Transwell migration assays showed that the migration rate of Cbl-b shRNA clones in response to RANKL stimulation was significantly higher than that of non-silencing controls (Figure 3F). On the contrary, the migration rate of Cbl-b overexpression clones in response to RANKL stimulation was significantly lower than that of controls (Supplementary Figure S1).

**Figure 4:**
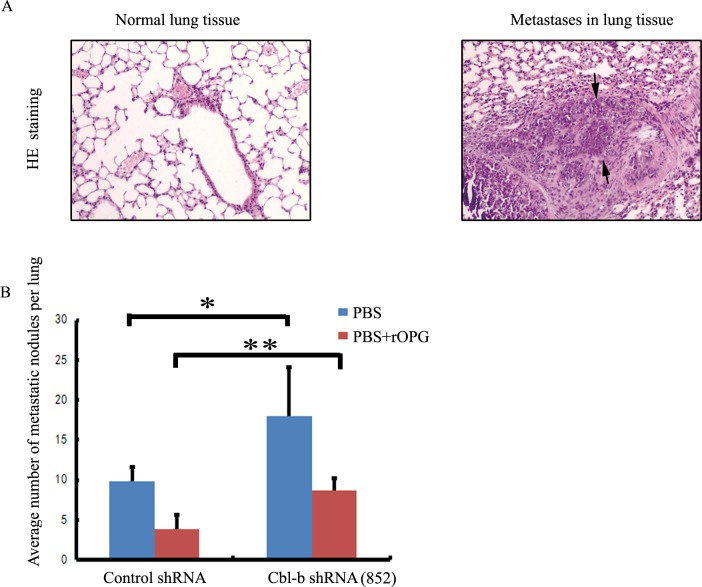
Cbl-b inhibited RANKL-induced metastasis of breast cancer **A.** After injection of MDA-MB-231 control shRNA cells or Cbl-b shRNA cells, representative hematoxylin and eosin (HE) staining of metastatic tumors and normal lung tissues are shown. Arrows show typical examples of lung metastases. Magniﬁcation × 100. **B.** Number of metastases in the lungs of mice (n=5 per group) 8 weeks after tail-vein injection of control shRNA or Cbl-b shRNA cells, with 20 mg of rOPG or an equal volume of PBS. Mean nodule values per lung are shown. Treatment details are given in the Methods section. Results are expressed as the mean ±SD. *p < 0.05, **p < 0.01 indicate significant differences compared with the control.

To evaluate the *in vivo* effects of Cbl-b depletion on metastasis, MDA-MB-231 Cbl-b shRNA cells or non-silencing control cells were injected into lateral tail vein of female nude mice separately or combined with OPG or PBS, and mice were killed 8 weeks later. Immunohistochemical analysis showed that mice treated with Cbl-b shRNA cells had a markedly higher rate of development of lung metastatic foci than those treated with non-silencing control cells (Figure 4A and 4B).

These results indicated that Cbl-b negatively regulates RANKL-induced breast cancer cell migration and inhibits RANKL-induced metastasis of breast cancer.

### Cbl-b is a negative regulator of the RANKL/RANK pathway and RANK expression during breast cancer cell migration

**Figure 5:**
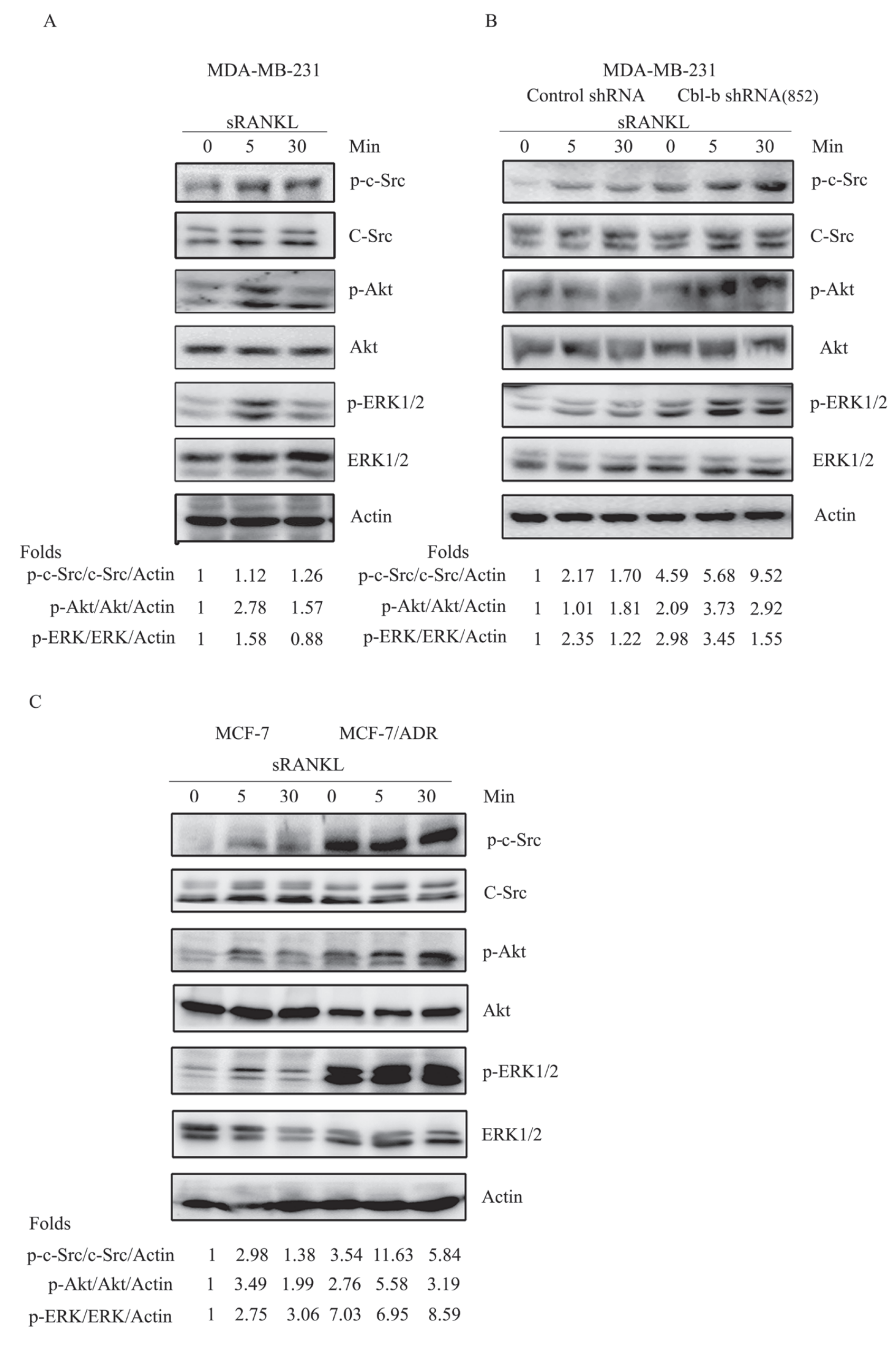
Cbl-b negatively regulated the RANKL/RANK pathway **A.** MDA-MB-231 cells were incubated with sRANKL for the indicated time intervals, and p-c-Src/c-Src, p-Akt/Akt, p-ERK/ERK and β-actin (Actin) were examined by western blotting. **B.** MDA-MB-231Cbl-b shRNA clones and control shRNA clones were treated with or without 2 μg/ml of sRANKL for the indicated times. The activation of c-Src, ERK and Akt was analyzed by western blotting. **C.** MCF-7 and MCF-7/ADR were treated with or without 2 μg/ml of sRANKL for the indicated times. The activation of c-Src, ERK and Akt was analyzed by western blotting.

To elucidate the exact mechanism underlying the regulation of the RANKL/RANK pathway by Cbl-b, we performed western blot analyses, which showed that c-Src, Akt and ERK1/2 were activated in response to RANKL stimulation (Figure 5A). Furthermore, a significantly enhanced phosphorylation of c-Src, Akt and ERK was detected in both MDA-MB-231 Cbl-b shRNA cells and Cbl-b low-expressing MCF-7/ADR, compared to non-silencing controls and MCF-7 (Figure 5B and 5C). In addition, to further confirm our finding, we performed transient transfection of MCF-7 cells with pcDNA3.1 plasmid that includes the full-length cDNA for Cbl-b, and also with a second independent shRNA (414) against Cbl-b, separately. The result showed that RNA silencing expression of Cbl-b enhanced the activation of c-Src, Akt and ERK1/2 induced by RANKL, while, over-expressing Cbl-b inhibited the activation of those signaling molecules (Supplementary Figure S1).

Similarly, as previously reported [29], c-Src lies upstream of ERK and Akt, which suggests that Cbl-b inhibited RANKL-induced breast cancer cell migration via the c-Src/Akt and c-Src/ERK pathways. It should be mentioned that the expression of c-Src in MDA-MB-231 and MCF-7 cells was not affected by treatment with RANKL, suggesting that Cbl-b did not interact directly with c-Src as a ubiquitin ligase during the course of RANKL-induced breast cancer cell migration.

**Figure 6:**
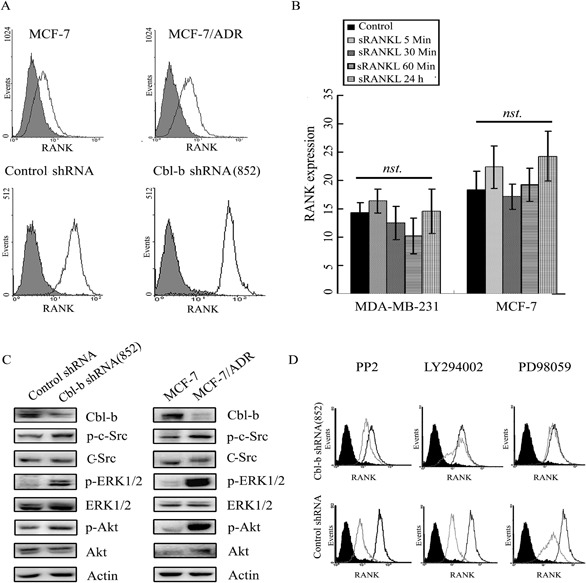
Cbl-b negatively regulated RANK expression by inhibiting p-Src, p-Akt, and p-ERK levels **A.** RANK expression on the surface of MCF-7, MCF-7/ADR, MDA-MB-231Cbl-b shRNA clones and control shRNA clones was examined by ﬂow cytometry; isotype control staining is shown in grey. **B.** After treatment with RANKL for the indicated times, RANK expression on the surface of MDA-MB-231 and MCF-7 cells was examined by ﬂow cytometry. Results are expressed as the mean ± SD. Data represent the results of at least three independent experiments. **C.** Levels of Cbl-b, p-Src/Src, p-ERK/ERK, p-Akt/Akt and β-actin (Actin) in MCF-7, MCF-7/ADR, MDA-MB-231 control shRNA, and MDA-MB-231 Cbl-b shRNA cells were examined by western blotting. **D.** MDA-MB-231 control shRNA, and MDA-MB-231 Cbl-b shRNA cells were treated with or without PP2 (Src inhibitor, 10 μM), LY294002 (PI3K inhibitor, 25 μM), or PD98059 (ERK inhibitor, 20 μΜ) for 24 h, and RANK expression on the surface of cells was detected by ﬂow cytometry. The black area indicates isotype control staining, the black lines indicate untreated experimental cells, and the grey lines indicate treated experimental cells.

Since receptor overexpression, such as EGFR, can result in sustained activation of downstream signaling [20], and we previously reported that the ubiquitin ligase inhibitor PS-341 enhances RANK expression in MDA-MB-231 cells, we examined the effect of Cbl-b on RANK expression. Flow cytometry analysis showed higher expression levels of RANK in MCF-7/ADR cells (low Cbl-b expression) than in MCF-7 cells. Similar results were obtained in MDA-MB-231 Cbl-b shRNA cells and MCF-7 Cbl-b shRNA cells compared to their controls (Figure 6A, Supplementary Figure S1). While, the RANK expression of Cbl-b overexpression clones decreased compared to controls (Supplementary Figure S1). These results indicated that Cbl-b inhibited the expression of the RANK protein. In addition, no significant differences in RANK expression were observed between MDA-MB-231 or MCF-7 cells and their parental controls (Figure 6B) after treatment with RANKL for the indicated time points. These results suggested that Cbl-b did not interact directly with RANK in RANKL-induced breast cancer cell migration. Western blot analysis showed that the basal levels of p-Src, p-Akt and p-ERK were significantly higher in MCF-7/ADR clones than in MCF-7 cells, and a similar result was obtained in MDA-MB-231Cbl-b shRNA cells compared with non-silencing controls (Figure 6C). Treatment with either the c-Src inhibitor PP2, the PI3-K inhibitor LY294002 or the MEK inhibitor PD98059 significantly down-regulated RANK expression in MDA-MB-231Cbl-b shRNA clones (Figure 6D). These results indicated that p-Src/p-ERK and p-Src/p-Akt positively regulate RANK expression.

We further evaluated the expression of p-ERK and p-Akt and the correlation between p-ERK and p-Akt and RANK in breast cancer tissues. As shown in Table 3, RANK expression was significantly positively correlated with p-ERK and p-Akt, which confirmed that p-Src/p-ERK and p-Src/p-Akt are positive regulators of RANK expression.

Taken together, our results suggested that Cbl-b negatively regulates the RANKL/RANK pathway in association with the migration of breast cancer cells, and Cbl-b negatively regulates RANK expression by inhibiting p-Src, p-Akt, and p-ERK.

**Table 3:** Correlation estimates between expression of p-ERK, P-Akt and RANK, investigated by Pearson's correlation analysis

	RANK	p-ERK	p-Akt
**RANK**	1		
**p-ERK**	0.260**	1	
**p-Akt**	0.307**	0.524**	1

** Correlation is significant at the 0.01 level (two-tailed).

## DISCUSSION

The RANKL/RANK pathway plays an essential role in osteoclast maturation and activation during bone metastasis. Denosumab, an antibody against RANKL, was developed for the treatment of bone metastasis in cancer patients and has shown good efficacy in the clinical setting [30-32]. Preclinical observations suggest that RANKL promotes bone and lung metastasis via the direct pro-metastatic effects of RANKL on RANK expressing breast cancer cells [9] independently of osteoclasts, implying that the RANKL/RANK pathway may also play a role in non-bone metastasis. However, whether RANK expression affects the progression and survival of breast cancer patients remains unclear. Although some clinical studies have confirmed the *in vitro* studies' results, certain contradictory findings have been reported. In the present study, RANK was expressed in 51.3% of breast cancer tissues, and RANK expression was not associated with poor prognosis, which was an unpredictable result. Since breast cancer is widely recognized as a heterogeneous disease, other factors may exist and affect the prognosis of RANK-expressing patients. Since Cbl-b is an essential regulator of the RANKL/RANK pathway in osteoclasts and dendritic cells, we performed stratified analysis to explore the effect of Cbl-b on the prognosis of RANK-expressing patients. Our results showed that in RANK-positive individuals, Cbl-b expression was significantly inversely correlated with the incidence of metastasis, including bone and visceral metastasis. Survival analysis showed that Cbl-b expression was an independent predictor of favorable DFS in RANK-positive patients but not in RANK-negative individuals. To the best of our knowledge, this is the first study showing that Cbl-b exerts a protective effect against disease progression in RANK-expressing breast cancer. Our findings indicate that combined detection of RANK and Cbl-b may add signiﬁcant prognostic value to pathologic staging, histologic grade, and standard clinical molecular markers.

Since metastasis is a complex process, and the active migration of tumor cells is a prerequisite for tumor cell invasion and metastasis, a migration assay was performed to explore the mechanism underlying the protective effect of Cbl-b against metastasis of RANK-expressing breast cancer. The results of cellular level experiments presented here showed that in Cbl-b shRNA cells and MCF-7/ADR cells, which natively express low levels of Cbl-b, RANKL-induced breast cancer cell migration was significantly increased compared with the respective controls. In addition, animal experiments showed that mice infused with Cbl-b shRNA cells had a higher rate of lung metastasis than those with control shRNA cells. These results suggested that Cbl-b inhibited breast cancer metastasis by negatively regulating RANKL-induced breast cancer cell migration. Regarding the exact mechanism underlying the Cbl-b mediated regulation of the RANKL/RANK pathway during breast cancer cell migration, our previous report and those of other groups showed that RANKL promotes breast cancer cell migration through c-Src-dependent Akt and ERK pathways [29, 33]. The present study further showed that Cbl-b inhibited breast cancer cell migration by suppressing the activation of c-Src, ERK and Akt, indicating that Cbl-b inhibited RANKL-induced breast cancer cell migration via the Src-ERK/Akt pathway.

Meanwhile, the present data demonstrated that RANK expression was significantly upregulated in Cbl-b shRNA cells and MCF-7/ADR cells, compared with control shRNA cells and MCF-7 cells, respectively, and was down-regulated in Cbl-b overexpression MCF-7 clones. To date, little is known about the mechanism by which different stimuli regulate RANK expression. Hie et al. reported that M-CSF-induced RANK expression is mediated by the activation of ERK in bone marrow-derived monocyte/macrophage cells [34]. Tang et al. recently reported that hypoxia up-regulates RANK expression via the PI3K/Akt-HIF-1a pathway in MDA-MB-231 and MCF-7 cells [35]. In the present study, inhibition of c-Src, ERK or Akt using specific inhibitors reversed the up-regulation of RANK expression in Cbl-b shRNA cells and MCF-7/ADR cells. Analysis of clinical specimens showed a significant positive correlation between p-ERK or p-Akt and RANK expression, confirming the positive regulation of ERK and Akt activation by RANK. Since c-Src functions upstream of both ERK and Akt in the RANKL/RANK pathway [29], these results suggested that RANK expression is regulated by the c-Src/ERK and c-Src/Akt pathways.

Additionally, the results of the present study indicated that RANK and c-Src expression in breast cancer cells did not change significantly in response to treatment with RANKL. This suggested that the inhibition of RANK expression and the RANKL/RANK pathway by Cbl-b was not mediated by ubiquitination of RANK or c-Src. Cbl-b may negatively regulate RANKL/RANK signaling via its adaptor function, although further investigation is necessary to elucidate the exact mechanism.

**Figure 7:**
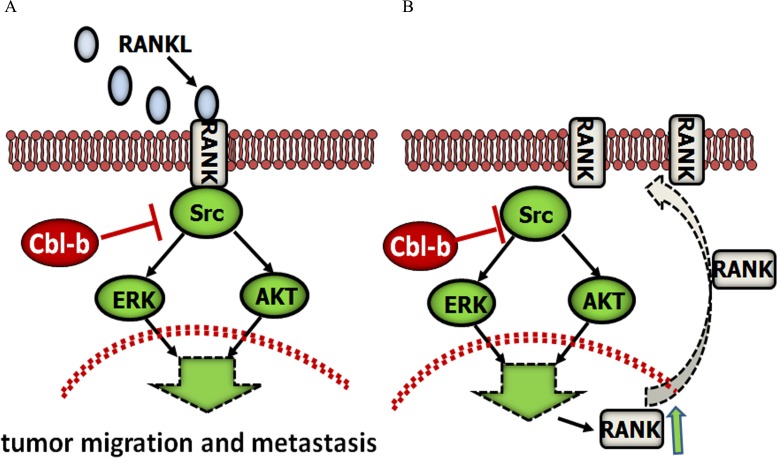
Proposed model for the role of Cbl-b in RANKL-induced breast cancer cell migration and metastasis Cbl-b inhibited RANKL-induced breast cancer cell migration and metastasis, and Cbl-b down-regulated RANK protein expression by negatively regulating the Src-Akt/ERK pathway.

In summary, this is the first report showing that Cbl-b acts as a “switch protein”, negatively regulating the RANKL/RANK pathway (Figure 7). Cbl-b expression status played a significant role in the metastasis and prognosis of RANK-expressing patients, suggesting that the combined analysis of Cbl-b and RANK as biomarkers could be helpful for the characterization of breast cancer patients. The present findings provide insight into the biology of breast cancer metastasis, and may help guide individualized patient management. However, large multi-institutional prospective studies are necessary to confirm our observations, and the exact molecular regulatory mechanism should be examined in future studies.

## PATIENTS, MATERIALS AND METHODS

### Reagents

Recombinant sRANKL and recombinant human OPG (rOPG) were purchased from Cytolab/Peprotech Asia (NJ, USA). The PI3K inhibitor LY294002 and the Src inhibitor PP2 were obtained from Sigma (St. Louis, MO, USA). The ERK inhibitor PD98059 was purchased from Promega (Madison, WI, USA).

### Patients and tissue samples

A total of 300 breast cancer samples obtained from patients who underwent surgery at our hospital between 1997 and 2010 were included in the analysis. The present study was approved by the Human Ethics Review Committee of the First Hospital of China Medical University (NO.2011 [11]). Informed consent was obtained from all patients in accordance with the Declaration of Helsinki and its later revision. All patients had been treated according to guidelines, including adjuvant chemotherapy, irradiation or endocrine therapy according to lymph node metastasis and hormone receptor status. DFS was deﬁned as the time from the date of diagnosis to the date of distant metastases and/or cancer-related death. BCSS was deﬁned as the time from the date of diagnosis to the date of cancer related death.

### Immunohistochemistry

Formalin-ﬁxed parafﬁn-embedded tumor specimens were collected from the Department of Pathology at the First Hospital of China Medical University. Immunohistochemical staining was performed using the following antibodies: rabbit anti-RANK (sc-52951, 1:500, Santa Cruz Biotechnology, Santa Cruz, USA), anti-Cbl-b (sc-376409, 1:250, Santa Cruz Biotechnology), p-ERK (1:1000, Cell Signaling Technology, Danvers, MA, USA) and p-Akt (1:1000; Cell Signaling) using the biotin-streptavidin method (UltraSensitive S-P kit; Maixin-Bio, Shanghai, China) as described previously [36]. The evaluation of immunohistochemistry results was performed independently by two observers who had no prior knowledge of the clinical information or pathological parameters. The immunoreactivity was scored based on both intensity of staining (negative = 0, weak = 1, moderate = 2, strong = 3) and percentage of positive tumor cells (<10% = 0, 10%–50% = 1, >50% = 2) as reported previously. The ﬁnal score was calculated by multiplying the single scores to obtain the intensity and percentage of positive cells (range from 0 to 6) [36, 37]. The median expression score for both RANK and Cbl-b was 2, and this was used as a cut-off value, with a score of at least 2 considered positive.

### Cell culture

The breast cancer cell lines MDA-MB-231 and MCF-7 were obtained from the Type Culture Collection of the Chinese Academy of Sciences (Shanghai, China). The human breast cancer multi-drug resistant cell variant MCF-7/ADR was obtained from Fourth Military Medical University (Xi'an, China). MDA-MB-231 cells were grown in Leibovitz L-15 medium (Gibco-Invitrogen, CA, USA) containing 10% FBS; MCF-7 and MCF-7/ADR cells were cultured in RPMI-1640 (Gibco-Invitrogen) containing 10% fetal bovine serum (FBS), according to the manufacturer's instructions. For MCF-7/ADR cells, the medium additionally contained 1 μg/mL adriamycin to maintain the drug resistance phenotype.

### Surface RANK expression analysis

Surface RANK expression was determined by ﬂow cytometry as previously described [29]. The following antibodies were used: mouse anti-RANK (R&D, USA) or isotype control (R&D), FITC-conjugated anti-mouse secondary antibody (Santa Cruz Biotechnology).

### Western blot analysis

Western blotting was performed as previously described [29], using the following antibodies: mouse anti-Cbl-b, mouse anti-c-Src, and rabbit anti-β-actin antibodies were obtained from Santa Cruz Biotechnology; rabbit anti-Akt, anti-p-Akt (Ser473), anti-ERK1/2, anti-p-ERK1/2 (Thr202/Tyr204) and anti-p-Src (Y416) antibodies were obtained from Cell Signaling Technology, mouse anti-RANK antibodies were obtained from R&D, USA. Proteins were visualized using the enhanced chemiluminescence reagent (SuperSignal Western Pico Chemiluminescent Substrate; Pierce, Rockford, IL, USA) and signals were quantitated using NIH Image J software.

### Transwell migration assay

The Transwell migration assay was performed as previously described [29]. A 24-well chemotaxis chamber (8-μm pore size; Corning, USA) was used for this experiment. Briefly, 4×10^4^ cells were loaded onto the upper well of the Transwell chamber and medium containing sRANKL, the respective inhibitors or DMSO was added to the bottom well. The plates were incubated for 16 h at 37°C. Then, the porous inserts were carefully removed with a cotton swab and the cells that had traversed the membrane were ﬁxed, stained with Trypan Blue and counted.

### Transfection with short hairpin RNA (shRNA)

As previously described by our group [38], two sets of synthetic oligonucleotides encompassing the sense and antisense target sequences of human Cbl-b (Human Cbl-b sepcific sequence: 5′- GATCCCGTTTCCGGTTAAGTTGCACTCGTTCAA GAGACGAGTGCAACTTAACCGGAAATTTTTT CCAAA-3′ and 5′-AGCTTTTGGAAAAAA TTTCCGGTTAAGTTGCACTCGTCTCTTG AACGAGTGCAACTTAACCGGA AAGG-3′ for Cbl-b (852); 5′- GATCCCGGACAGACGGAATCTCACATT GATAT CCGTGTGAGATTCCGTC TGTCCTTTTTTCCAAA - 3′ and 5′- AGCTTTTGGAA AAAAGGA CAGACGG AATCTCACACGGATATCAATGTGAGA TTCCG TCTGTCCGG-3′ for Cbl-b (414), and one set of non-silencing control: 5′-GATCCCGTTCTCCGAA CGTGTCACGTTTGATATCCGA CGTGACACGTTCGGAGAATTTTTTCCAAA-3′ and 5′-AGCTTTTGGAAAAAA TTCTCCGAACGTGTCACGTCGGATATCZAA CGTGACACGTTCGGAGAACGG-3′ were phosphorylated with T4 kinase (Takara, Tokyo, Japan), annealed, and ligated into the Bam-HI/Hind III-cleaved backbone of pRNA-U6.1/Neo (Genscript, Piscataway, NJ, USA). shRNA (852)-expressing vectors were transfected into MDA-MB-231, and shRNA (414)-expressing vectors into MCF-7 cells, using Lipofectamine™ 2000 reagent (Invitrogen, Carlsbad, CA, USA), separately. Forty-eight hours later, transient transfected MCF-7 cells were used to perform next experiments, and stably transfected MDA-MB-231cells were screened with G418 (Invitrogen). The expression of Cbl-b was verified by western blotting. Stably transfected MDA-MB-231cell lines expressing Cbl-b at <10% of the level of endogenous Cbl-b were used for subsequent experiments.

### Transient transfection of plasmid constructs

MCF-7 cells were plated in six-well plates and cultured in drug-free medium. At 90-95% conﬂuence, cells were washed twice with PBS and incubated in 2 ml of RPMI 1640 medium without antibiotics. the pcDNA encodes Cbl-b was obtained from Dr. Stanlely Lipkowitz (National Naval Medical Center, Bethesda, MD) and subcloned into pcDNA expressing vector. Cbl-b/pcDNA 3.1(Invitrogen) was transiently transfected employing the Lipofectamine 2000 reagent (Invitrogen, Inc, Carlsbad, CA, USA). Cells transfected with the pcDNA3.1 vector alone served as the negative control. Forty-eight hours later, cells were used to perform next experiments.

### *In vivo* tail-vein metastasis assay

Animal experiments were carried out in accordance with the European Guidelines for the Care and Use of Laboratory Animals, Directive 2010/63/UE. Female bal/bc nude (nu/nu) mice, 4 weeks of age, were purchased from SLAC Animal Center (Shanghai, China). A total of 1×10^6^ cells (in 200 μL PBS) of MDA-MB-231 non-silencing control cells or MDA-MB-231Cbl-b shRNA cells were injected into the caudal vein of nude mice. On days 1, 2, and 3, either 20 mg of OPG or an equal volume of PBS was injected into the caudal vein of nude mice. Eight weeks after injection, all mice were killed and their lungs were fixed in 10% formalin. Microscopic quantiﬁcation of lung foci was performed on representative serial cross-sections of formalin-ﬁxed, parafﬁn-embedded lungs stained with hematoxylin and eosin (HE).

### Statistical analysis

Statistical analyses were performed using SPSS18.0 for Windows (Chicago, IL, USA). All experiments were repeated at least three times. Data were expressed as the mean ± standard deviation (SD). Statistical significance was determined by Student's t-test. All tests were one-sided and *p* < 0.05 was considered to be statistically significant. The association of staining intensity with clinic-pathological patterns was assessed using the Chi-square test. Pearson's correlation coefficient was used to calculate the correlation between RANK score, p-ERK score, and p-Akt score. The log-rank test and the Kaplan-Meier method were used for analysis of patient survival. Univariate and multivariate analyses were performed according to the Cox proportional hazards model. *p* < 0.05 was considered significant.

## SUPPLEMENTARY MATERIAL FIGURE AND TABLE



## References

[1] Nakagawa N, Kinosaki M, Yamaguchi K, Shima N, Yasuda H, Yano K, Morinaga T, Higashio K (1998). RANK is the essential signaling receptor for osteoclast differentiation factor in osteoclastogenesis. Biochemical and biophysical research communications.

[2] Anderson DM, Maraskovsky E, Billingsley WL, Dougall WC, Tometsko ME, Roux ER, Teepe MC, DuBose RF, Cosman D, Galibert L (1997). A homologue of the TNF receptor and its ligand enhance T-cell growth and dendritic-cell function. Nature.

[3] Josien R, Wong BR, Li HL, Steinman RM, Choi Y (1999). TRANCE, a TNF family member, is differentially expressed on T cell subsets and induces cytokine production in dendritic cells. Journal of immunology (Baltimore, Md : 1950).

[4] Fata JE, Kong YY, Li J, Sasaki T, Irie-Sasaki J, Moorehead RA, Elliott R, Scully S, Voura EB, Lacey DL, Boyle WJ, Khokha R, Penninger JM (2000). The osteoclast differentiation factor osteoprotegerin-ligand is essential for mammary gland development. Cell.

[5] Mikami S, Katsube K, Oya M, Ishida M, Kosaka T, Mizuno R, Mochizuki S, Ikeda T, Mukai M, Okada Y (2009). Increased RANKL expression is related to tumour migration and metastasis of renal cell carcinomas. Journal of Pathology.

[6] Jones DH, Nakashima T, Sanchez OH, Kozieradzki I, Komarova SV, Sarosi I, Morony S, Rubin E, Sarao R, Hojilla CV, Komnenovic V, Kong YY, Schreiber M (2006). Regulation of cancer cell migration and bone metastasis by RANKL. Nature.

[7] Chen L-M, Kuo C-H, Lai T-Y, Lin Y-M, Su C-C, Hsu H-H, Tsai F-J, Tsai C-H, Huang C-Y, Tang C-H (2011). RANKL increases migration of human lung cancer cells through intercellular adhesion molecule-1 up-regulation. Journal of Cellular Biochemistry.

[8] Santini D, Perrone G, Roato I, Godio L, Pantano F, Grasso D, Russo A, Vincenzi B, Fratto ME, Sabbatini R, Della Pepa C, Porta C, Del Conte A (2011). Expression pattern of receptor activator of NFκB (RANK) in a series of primary solid tumors and related bone metastases. Journal of Cellular Physiology.

[9] Palafox M, Ferrer I, Pellegrini P, Vila S, Hernandez-Ortega S, Urruticoechea A, Climent F, Soler MT, Munoz P, Vinals F, Tometsko M, Branstetter D, Dougall WC (2012). RANK induces epithelial-mesenchymal transition and stemness in human mammary epithelial cells and promotes tumorigenesis and metastasis. Cancer research.

[10] Tan W, Zhang W, Strasner A, Grivennikov S, Cheng JQ, Hoffman RM, Karin M (2011). Tumour-infiltrating regulatory T cells stimulate mammary cancer metastasis through RANKL-RANK signalling. Nature.

[11] Santini D, Schiavon G, Vincenzi B, Gaeta L, Pantano F, Russo A, Ortega C, Porta C, Galluzzo S, Armento G, La Verde N, Caroti C, Treilleux I (2011). Receptor Activator of NF-kB (RANK) Expression in Primary Tumors Associates with Bone Metastasis Occurrence in Breast Cancer Patients. PLoS One.

[12] Pfitzner BM, Branstetter D, Loibl S, Denkert C, Lederer B, Schmitt WD, Dombrowski F, Werner M, Rudiger T, Dougall WC, von Minckwitz G (2014). RANK expression as a prognostic and predictive marker in breast cancer. Breast Cancer Research and Treatment.

[13] Sanger N, Ruckhaberle E, Bianchini G, Heinrich T, Milde-Langosch K, Muller V, Rody A, Solomayer EF, Fehm T, Holtrich U, Becker S, Karn T (2014). OPG and PgR show similar cohort specific effects as prognostic factors in ER positive breast cancer. Molecular Oncology.

[14] Owen S, Ye L, Sanders AJ, Mason MD, Jiang WG (2013). Expression profile of receptor activator of nuclear-kappaB (RANK), RANK ligand (RANKL) and osteoprotegerin (OPG) in breast cancer. Anticancer Research.

[15] Zhang H, Wu C, Matesic LE, Li X, Wang Z, Boyce BF, Xing L (2013). Ubiquitin E3 ligase Itch negatively regulates osteoclast formation by promoting deubiquitination of tumor necrosis factor (TNF) receptor-associated factor 6. The Journal of biological chemistry.

[16] Takayanagi H, Ogasawara K, Hida S, Chiba T, Murata S, Sato K, Takaoka A, Yokochi T, Oda H, Tanaka K, Nakamura K, Taniguchi T (2000). T-cell-mediated regulation of osteoclastogenesis by signalling cross-talk between RANKL and IFN-gamma. Nature.

[17] Nakajima A, Sanjay A, Chiusaroli R, Adapala NS, Neff L, Itzsteink C, Horne WC, Baron R (2009). Loss of Cbl-b increases osteoclast bone-resorbing activity and induces osteopenia. Journal of bone and mineral research : the official journal of the American Society for Bone and Mineral Research.

[18] Yamaguchi N, Yokota M, Taguchi Y, Gohda J, Inoue J (2012). cIAP1/2 negatively regulate RANKL-induced osteoclastogenesis through the inhibition of NFATc1 expression. Genes to cells : devoted to molecular & cellular mechanisms.

[19] Lee Y, Hyung SW, Jung HJ, Kim HJ, Staerk J, Constantinescu SN, Chang EJ, Lee ZH, Lee SW, Kim HH (2008). The ubiquitin-mediated degradation of Jak1 modulates osteoclastogenesis by limiting interferon-beta-induced inhibitory signaling. Blood.

[20] Weissman AM (1997). Regulating protein degradation by ubiquitination. Immunology today.

[21] Sévère N, Dieudonné FX, Marie PJ (2013). E3 ubiquitin ligase-mediated regulation of bone formation and tumorigenesis. Cell Death and Disease.

[22] Zhang L, Teng Y, Zhang Y, Liu J, Xu L, Qu J, Hou K, Liu Y, Qu X (2012). Proteasome inhibitor bortezomib (PS-341) enhances RANKL-induced MDA-MB-231 breast cancer cell migration. Molecular medicine reports.

[23] Yan S, Qu X, Xu Ca, Zhu Z, Zhang L, Xu L, Song N, Teng Y, Liu Y (2012). Down-regulation of Cbl-b by bufalin results in up-regulation of DR4/DR5 and sensitization of TRAIL-induced apoptosis in breast cancer cells. Journal of Cancer Research and Clinical Oncology.

[24] Qu X, Zhang Y, Li Y, Hu X, Xu Y, Xu L, Hou K, Sada K, Liu Y (2009). Ubiquitin ligase Cbl-b sensitizes leukemia and gastric cancer cells to anthracyclines by activating the mitochondrial pathway and modulating Akt and ERK survival signals. FEBS Letters.

[25] Qu X (2004). Negative regulation of Fc RI-mediated mast cell activation by a ubiquitin-protein ligase Cbl-b. Blood.

[26] Qiao G, Li Z, Molinero L, Alegre ML, Ying H, Sun Z, Penninger JM, Zhang J (2008). T-cell receptor-induced NF-kappaB activation is negatively regulated by E3 ubiquitin ligase Cbl-b. Molecular and cellular biology.

[27] Duan L, Raja SM, Chen G, Virmani S, Williams SH, Clubb RJ, Mukhopadhyay C, Rainey MA, Ying G, Dimri M, Chen J, Reddi AL, Naramura M (2011). Negative regulation of EGFR-Vav2 signaling axis by Cbl ubiquitin ligase controls EGF receptor-mediated epithelial cell adherens junction dynamics and cell migration. The Journal of biological chemistry.

[28] Okabe S, Tauchi T, Ohyashiki K, Broxmeyer HE (2006). Stromal-cell-derived factor-1/CXCL12-induced chemotaxis of a T cell line involves intracellular signaling through Cbl and Cbl-b and their regulation by Src kinases and CD45. Blood cells, molecules & diseases.

[29] Zhang L, Teng Y, Zhang Y, Liu J, Xu L, Qu J, Hou K, Yang X, Liu Y, Qu X (2012). C-Src-mediated RANKL-induced breast cancer cell migration by activation of the ERK and Akt pathway. Oncology letters.

[30] Smith MR, Saad F, Coleman R, Shore N, Fizazi K, Tombal B, Miller K, Sieber P, Karsh L, Damiao R, Tammela TL, Egerdie B, Van Poppel H (2012). Denosumab and bone-metastasis-free survival in men with castration-resistant prostate cancer: results of a phase 3, randomised, placebo-controlled trial. Lancet.

[31] Iranikhah M, Wilborn TW, Wensel TM, Ferrell JB (2012). Denosumab for the prevention of skeletal-related events in patients with bone metastasis from solid tumor. Pharmacotherapy.

[32] Stopeck AT, Lipton A, Body JJ, Steger GG, Tonkin K, de Boer RH, Lichinitser M, Fujiwara Y, Yardley DA, Viniegra M, Fan M, Jiang Q, Dansey R (2010). Denosumab compared with zoledronic acid for the treatment of bone metastases in patients with advanced breast cancer: a randomized, double-blind study. Journal of Clinical Oncology.

[33] Tang ZN, Zhang F, Tang P, Qi XW, Jiang J (2011). RANKL-induced migration of MDA-MB-231 human breast cancer cells via Src and MAPK activation. Oncology reports.

[34] Hie M, Tsukamoto I (2011). Vitamin C-deficiency stimulates osteoclastogenesis with an increase in RANK expression. The Journal of nutritional biochemistry.

[35] Tang ZN, Zhang F, Tang P, Qi XW, Jiang J (2011). Hypoxia induces RANK and RANKL expression by activating HIF-1alpha in breast cancer cells. Biochemical and biophysical research communications.

[36] Zhang L, Teng Y, Zhang Y, Liu J, Xu L, Qu J, Hou K, Yang X, Liu Y, Qu X (2012). Receptor activator for nuclear factor kappa B expression predicts poor prognosis in breast cancer patients with bone metastasis but not in patients with visceral metastasis. Journal of clinical pathology.

[37] Broustas CG, Ross JS, Yang Q, Sheehan CE, Riggins R, Noone AM, Haddad BR, Seillier-Moiseiwitsch F, Kallakury BV, Haffty BG, Clarke R, Kasid UN (2010). The proapoptotic molecule BLID interacts with Bcl-XL and its downregulation in breast cancer correlates with poor disease-free and overall survival. Clinical cancer research: an official journal of the American Association for Cancer Research.

[38] Feng D, Ma Y, Liu J, Xu L, Zhang Y, Qu J, Liu Y, Qu X (2013). Cbl-b enhances sensitivity to 5-fluorouracil via EGFR- and mitochondria-mediated pathways in gastric cancer cells. International journal of molecular sciences.

