# Supplementary material for: The E3 ubiquitin ligase Cbl-b improves the prognosis of RANK positive breast cancer patients by inhibiting RANKL-induced cell migration and metastasis
Source: Oncotarget. 2015 Jun 8;6(26):22918–33. doi: 10.18632/oncotarget.4382 (PMC4673209; doi:10.18632/oncotarget.4382)
Supplement: Supplementary file 1 [file oncotarget-06-22918-s001.pdf]

The E3 ubiquitin ligase Cbl-b improves the prognosis of RANK positive breast cancer patients by inhibiting RANKL-induced cell migration and metastasis

Supplementary Material

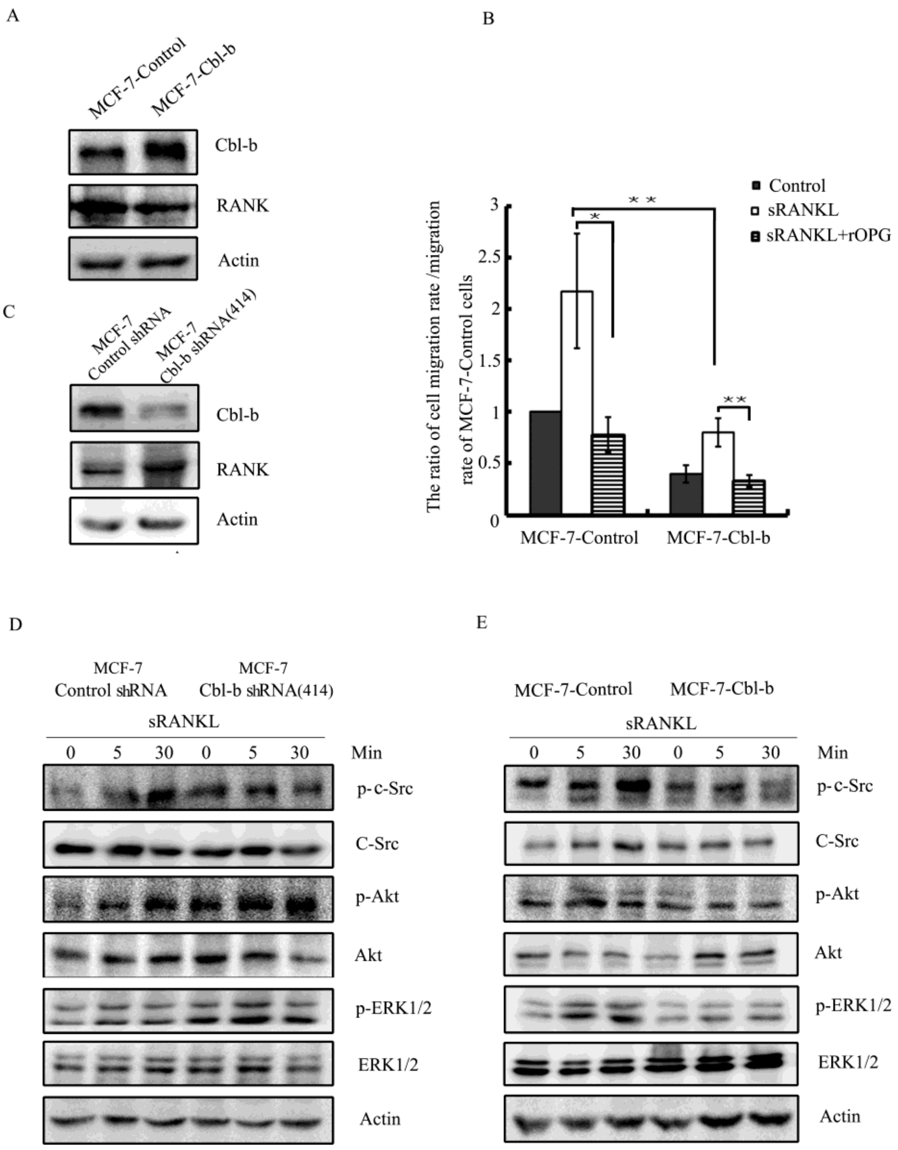

Supplementary Figure S1; Cbl-b negatively regulated RANK expression and RANKL/RANK pathway

(A) Western blot analysis showing the effect of overexpression of Cbl-b in MCF-7

clones. RANK expression was examined by western blotting.

(B) MCF-7 control and Cbl-b overexpression clones were incubated with sRANKL (2 μg/ml) with or without rOPG (10 μg/ml), and in vitro migration activity was measured with the Transwell assay. The data represent the results of at least three independent experiments. * $p<0.05$, ** $p<0.01$ indicate significant difference compared with the control.

(C) Western blot analysis showing the effect of shRNA against Cbl-b in MCF-7 clones. RANK expression was examined by western blotting.

(D) MCF-7 Control shRNA and MCF-7 Cbl-b shRNA were treated with or without 2 μg/ml of sRANKL for the indicated times. The activation of c-Src, ERK and Akt was analyzed by western blotting.

(E) MCF-7 control and Cbl-b overexpression clones were treated with or without 2 μg/ml of sRANKL for the indicated times. The activation of c-Src, ERK and Akt was analyzed by western blotting.

Table S1 : Correlation of RANK expression with clinic-pathological parameters in 300 breast cancer patients

Clinic-pathological parameters	n	RANK expression[†] (%)	*p*
**Histological type**			0.632
**invasive ductal carcinomas**	266	139(52.3)	
**invasive lobular carcinoma**	17	7(41.2)	
**others**	17	8(472.1)	
**Age (years)**			0.059
**≤35**	13	10(76.9)	
**＞35**	287	144(50.2)	
**Tumor size(cm)**			0.852
**≤2**	94	49(52.1)	
**＞2**	206	105(51.0)	
**$_{P}$N stage**			0.694
**0**	126	63(50.0)	
**1-3**	174	91(52.3)	
**Histology grade**			0.574
Ⅰ	30	14(46.7)	
Ⅱ+Ⅲ	236	123(52.1)	
**ER/PR status[††]**			0.151
**Negative**	133	74(55.6)	
**Positive**	165	78(47.3)	
**Missing**	2		
**HER2 status [§]**			**0.016**
**Negative**	188	88(46.8)	
**Positive**	104	64(61.5)	
**Missing**	8		
**Triple negative**			
**yes**	74	37(50.0)	0.763
**no**	223	116(52.0)	
**Missing**	3		
**Metastasis**			
**yes**	99	47(47.5)	0.348
**no**	201	107(53.2)	

P values shown in bold are statistically significant (two-sided, $p < 0.05$)

†, RANK expression was classified as "negative": arbitrary scale=0-1, and "positive": arbitrary scale=2-6, as described in materials and methods. ††, ER/PR status: Estrogen receptor or progesterone receptor status by immunohistochemistry (IHC); negative: ER and PR double negative; positive: ER or PR positive. §, HER2 status: HER2 positive status is IHC 3+ or fluorescence in situ hybridization (FISH) positive; HER2 negative status is IHC 0, 1+ or FISH negative, if. IHC 2+, FISH is applied to confirm the HER2 status.
